# Inter-rater reliability in clinical assessments: do examiner pairings influence candidate ratings?

**DOI:** 10.1186/s12909-020-02009-4

**Published:** 2020-05-11

**Authors:** Aileen Faherty, Tim Counihan, Thomas Kropmans, Yvonne Finn

**Affiliations:** National University of Ireland Galway, Galway, Ireland

**Keywords:** Clinical assessments, Reliability, Examiner variability, Examiner factors

## Abstract

**Background:**

The reliability of clinical assessments is known to vary considerably with inter-rater reliability a key contributor. Many of the mechanisms that contribute to inter-rater reliability however remain largely unexplained and unclear. While research in other fields suggests personality of raters can impact ratings, studies looking at personality factors in clinical assessments are few. Many schools use the approach of pairing examiners in clinical assessments and asking them to come to an agreed score. Little is known however, about what occurs when these paired examiners interact to generate a score. Could personality factors have an impact?

**Methods:**

A fully-crossed design was employed with each participant examiner observing and scoring. A quasi-experimental research design used candidate’s observed scores in a mock clinical assessment as the dependent variable. The independent variables were examiner numbers, demographics and personality with data collected by questionnaire. A purposeful sample of doctors who examine in the Final Medical examination at our institution was recruited.

**Results:**

Variability between scores given by examiner pairs (*N* = 6) was less than the variability with individual examiners (*N* = 12). 75% of examiners (*N* = 9) scored below average for neuroticism and 75% also scored high or very high for extroversion. Two-thirds scored high or very high for conscientiousness. The higher an examiner’s personality score for extroversion, the lower the amount of change in his/her score when paired up with a co-examiner; reflecting possibly a more dominant role in the process of reaching a consensus score.

**Conclusions:**

The reliability of clinical assessments using paired examiners is comparable to assessments with single examiners. Personality factors, such as extroversion, may influence the magnitude of change in score an individual examiner agrees to when paired up with another examiner. Further studies on personality factors and examiner behaviour are needed to test associations and determine if personality testing has a role in reducing examiner variability.

## Background

To become a competent physician, undergraduate medical students must be assessed not only on factual knowledge but also on communication and clinical skills. The reliability of clinical assessments to test these skills however, is known to be compromised by high levels of variability i.e. different results on repeated testing [1, 2].

Candidate variability, case variability (case specificity) and examiner variability all contribute to the overall variability of a clinical assessment. Candidate variability reflects the difference between candidates and in the absence of other variables (or error) candidate variability represents the true variability. Case specificity refers to the phenomenon that a candidate’s performance can vary from one case to the next due to differing levels of difficulty or content [2, 3]. Examiner variability refers to the fact that two examiners observing the same performance may award different scores. Many studies have shown that examiner variability is the most significant factor contributing to variability in clinical examinations [4, 5] and may even exceed the variability accounted for by differences in candidates [6]. Examiner variability is generally referred to as the degree of inter-examiner reliability, or the more commonly used term, inter-rater reliability. The level of inter-rater reliability which is deemed acceptable is a minimum of 0.6 with 0.8 being the gold standard (where 0 shows no relationship between two examiners scores and 1 is a perfect agreement) [7].

Variability in how examiners score candidates may be consistent, for example, an examiner who always marks candidates stringently (often referred to as a hawk) or an examiner who is consistently lenient (a dove) [3]. This kind of consistent examiner behavior can often be adjusted for when analyzing results. However, examiner behaviour may not *always* be so consistent and predictable.

Examiners in clinical assessments are subject to many forms of bias [8]. The ‘Halo effect’ refers to the phenomenon where an examiner’s overall first impression of a candidate (“*he seems like he knows his stuff*”) leads to failure to discriminate between discrete aspects of performance when awarding scores [9]. In addition, familiarity with candidates, the mood of the examiner and seeing information in advance have all also been found to affect examiners judgments [10–12]. Variability may result in a borderline candidate achieving a score in the pass range in one assessment and the same candidate failing a comparable assessment testing the same/similar competencies. In high stakes examinations, such as medical licensing examinations, this can have serious implications for both the candidate, the medical profession and even society in general. Moreover, pass/fail decisions are now increasingly being challenged [13].

While several strategies to reduce variability in clinical assessments have not been found to make any meaningful improvements to reliability [14], increasing the number of observations in an assessment (by involving more examiners in the observation of many performances) *has* [15]. In their evaluation of the mini-clinical exercise used in US medical licensing examinations, Margolis and colleagues stated that having a small number of raters rate an examinee multiple times was not as effective as having a larger number of raters rate the examinee on a smaller number of occasions and more raters enhanced score stability [6].

However, different raters are known to focus on different aspects of performance and groups are more likely to make unpopular decisions than single raters [16]. In addition, it was previously assumed that assessments conducted with others present (the overt condition) should lead to more reliable assessments [17]. Consequently, some institutions (including our own) have adopted the practice of pairing examiners and asking them to come to an agreed score rather than use individual raters. Little is known however, about what occurs when these paired examiners interact to generate a score.

In the field of Occupational Psychology, a meta-analysis conducted by Harari et al. looked at job performance ratings and found a relationship between the personality factors of the raters and the performance ratings given [18]. The ‘Big Five’ personality factors [19] (neuroticism, extroversion, openness to experience, agreeableness and conscientiousness) accounted for between 6 and 22% of the variance in performance ratings. Furthermore, other research in the areas of personality and Human Behaviour has shown that there is a relationship between the big five personality traits and the responsiveness of individuals to persuasion and influence strategies [20, 21]. Could an examiners personality make them more likely to influence or be influenced when examining in a pair?

In some of his work McManus hypothesized that personality may relate to examiner stringency [22], and there is evidence from one study that there is a correlation between personality type and examiner stringency [23]. While there are anecdotal reports of some medical-educators expressing concern that employing paired examiners could allow a dominant individual to unduly influence the decision process, this has not been well explored in the literature [16] and we found no studies that looked at the interaction between examiners in pairs.

### Summary of existing literature

Although the hawk-dove effect was described by Osler as far back as 1913 [23] its impact on the reliability of clinical examinations was only explored in recent years. In 1974 Fleming et al. described a major revision of the Membership of the Royal College of Physicians (MRCP) UK clinical examination and identified one examiner as a hawk [24]. There was a significantly lower pass rate in the group of candidates where this examiner examined compared with the remainder (46.3 and 66.0% respectively).

In 2006, an analysis of the reliability of the MRCP UK clinical examination that existed at that time, the Practical Assessment of Clinical Examination Skills (PACEs) exam, found that 12% of the variability in this examination was due to the hawk-dove effect [22]. Examiners were more variable than stations.

In 2008 Harasym et al. [25] found an even greater effect due to the hawk-dove phenomenon in an OSCE evaluating communication skills. Forty four percent of the variability in scores was due to differences in examiner stringency/leniency; over four times the variance due to student ability (10.3%).

As mentioned above, many types of rater-bias are known to be at play when human judgement comprises part of any assessment process (halo effect, the mood of the rater, familiarity with candidates, personality factors etc [8–11]). Yeates and colleagues in 2013 proposed three themes to explain how examiner-variability arises [26]. They termed these: differential salience (what was important to one examiner differed to another); criterion uncertainty (assessors’ conceptions of what equated to competence differed and were uncertain); information integration (assessors tend to judge in their own unique descriptive language forming global impressions rather than discrete numeric scores).

Govaerts suggests that some examiner-variability may simply arise from individual examiners’ peculiarities in approach and idiosyncratic judgements made as a result, of the interaction between social and cognitive factors [12].

Strategies to improve reliability in clinical assessments have ranged from increasing the number of items per station to implementing examiner training. Wilkinson et al. analysed examiners marks over a four-year period in New Zealand and found that while items-per-station increased over the 4 years, there was no correlation between items-per-station and the station inter-rater reliability [4]. Cook et al. [27] looked at the impact of examiner training and found no significant effect and while Holmboe et al. [28] showed that examiner training was associated with an increase in examiner stringency, this increase was inconsistent.

In a recent literature review on rater cognition in competency-based education Gauthier et al. [14] summarised the situation stating: “*attempts to address this variability problem by improving rating forms and systems, or by training raters, have not produced meaningful improvements”.*

In the field of psychology the Five-Factor Model of Personality (also referred to as the ‘Big Five’) has been proposed as an integrative framework for studying individual differences in personality and is among the most well accepted taxonomies of personality in the literature with wide application in different domains and across cultures due to its empirical validity [18, 20]. In this personality index, no single cut-off point separates those who “have” a particular personality trait from those who do not, rather individual scores represent degrees of each of the five main personality traits – neuroticism, extroversion, openness to experience, agreeableness and conscientiousness. Score results are usually expressed as a T score and can be further described as being very low, low, average, high and very high for each of the domains. The different personality traits are often associated with certain personal characteristics. Neuroticism has been linked to susceptibility to social influence strategies [20]. Extroversion has been found to be positively related to networking behaviours in organisations [29] and success in managerial and sales positions that require social interactions. Openness has been found to be the least susceptible personality trait to persuasion [21]. Other research has found agreeableness to be related to a tendency to favour positive social relationships and avoid conflict [30]. Employees who are high in conscientiousness generally display superior job performance as compared to employees who are lower in this trait [18].

In clinical examinations Finn et al. found examiner stringency was positively correlated with neuroticism and negatively with openness to experience [23]. The influence of examiner personality factors on scoring by examiner pairs has not been explored to date.

### Objectives

To analyse how an examiners’ marks vary from when s/he examines alone to when s/he examines in a pair.

To explore associations, if any, between examiner personality factors and examiner behaviour in scoring candidates.

To explore the usefulness of personality profiling in matching examiners to form an examiner pair.

### Research question

Do examiners’ marks for a given candidate differ significantly when that examiner marks independently compared with when that examiner marks in a pair?

Is there an association between examiner personality factors and examiner behaviour in marking candidate’s performances?

## Methods

### Design

A fully-crossed design was employed with each participant examiner observing and scoring recordings of candidates’ performances. A quasi-experimental research design was used. The dependent variable was candidate’s observed scores in a mock clinical assessment. The independent variables were examiner number (single or paired), examiner demographics and examiner personality. It should be noted that in this study the examin*ers* were the object of measurement, not the examin*ee*. There was no control group; examiner participants served as their own control i.e. control was exercised through more than one observation of the same phenomenon [31].

### Setting and characteristics of participants

The study population consisted of qualified medical doctors who examine in the final medical short-case examination at our institution. Participants were invited by email and each received a participant information leaflet, electronic consent form and demographic questionnaire.

### Description of all processes, interventions and comparisons

In the final medical examination at our school, medicine and surgery are assessed together in a short-case examination. Each candidate is assessed over 6 short-cases, a mixture of medical and surgical cases, each lasting 6 min using a real or simulated patient. Candidates are observed by pairs of examiners, usually a surgeon paired with a physician. After each candidates’ performance, examiners discuss and come to an agreed score using a domain-based marking sheet. Our data collection exercise was set up to mimic as closely as possible this real-world examination scenario using recordings of simulated patients.

Participants were stratified to mimic the examiner pairings usually employed (a surgeon with a physician). The participants did not assess a real students’ performance; instead we used video recordings of standardised student performances (using actors) that were previously created for the purposes of examiner training. We selected 3 videos as follows: one example each of a weak, average and good performance. Examiners were not aware what level of performance they would be watching. Different case types were selected (one medical, one surgical and one general medical/surgical) to avoid one examiner being more familiar than the other examiners with the content of the selected cases. Each participant viewed, initially on their own individual screens, the three recordings and graded them independently. The total possible score at each station was 50 marks – with ten marks each allocated to five separate domains; attitude and professionalism, communication skills, clinical skills, knowledge and lastly management. Our schools OSCE Management Information System Software – Qpercom Observe (Qpercom Ltd) was used to enter marks [32].

Utilising this software examiners were blinded to their individual scores of a given performance. When the examiners scored the performance across the individual five domains, the scores were on a slider and the examiner did not see what their resultant overall mark was from combining the 5 domains.

After the examiners had scored the videos independently there was a break for refreshments. Examiners then completed a validated 60 item personality questionnaire - the NEO Five Factor Index (NEO-FFI) [19]. This questionnaire was chosen given that the Five-Factor Model of Personality is among the most well accepted taxonomies of personality in the literature known for its empirical validity [18, 20].

After completing the personality questionnaire, examiners were moved to a neutral location and paired up with another examiner to review and discuss the same three performances again and this time devise a joint mark which was entered on OMIS. The order of the videos when watched as individual examiners compared with observing in pairs was counterbalanced to control for an order effect [33]. Blinding the participant as to the overall original scores given and changing the order of videos from the previous observation was particularly important to maintain internal validity. We looked for a correlation between the total amount of change in an examiners marks from when they examined individually to when they examined in a pair, and their personality scores.

### Statistical analysis

Data collected on candidate scores was analysed using the OMIS OSCE management software and SPSS 24 (IBM corp). Preliminary analyses confirmed that the data were not normally distributed and, therefore, non-parametric methods were employed in the statistical analysis. Descriptive statistics were generated using tables and charts. The OMIS OSCE management software allowed for psychometric analysis and provided support for generalisability analysis [34]. Generalisability is an inductive statistical method from the family of regression techniques – techniques which quantify relationships between variables to make predictions [3]. Generalisability theory assumes that variability is never simply arbitrary, but that a test score is determined by the condition of the ‘true’ construct being measured and ‘error factors’ which influence the score [3]. By analysing components of variance, Generalisability theory quantifies the impact that all the sources of error exert on the assessment score without multiple experiments [2]. The generalizability-coefficient as well as the absolute and relative SEM, 95% CI were also calculated using the statistical software program EDU-G 6.0 for Windows [35].

## Results

Fifty potential participants were contacted by email and invited to participate. Seventeen respondents accepted the invitation and 12 completed the study - 10 male and 2 female. They had an average of 13.6 years’ experience examining in the final-medical short-case examination at our institution. Two thirds were in posts that were combined clinical and academic. Two participants held formal qualifications in medical education.

### Variability

Table 1 shows the overall scores awarded by each examiner to the three candidates when examining alone and demonstrate considerable variability in examiners’ scores.

**Table 1:** Overall Scores for Good, Average and Weak Candidate comparing scores given by Single Examiners when examining alone and the agreed consensus score when in pairs. The middle column illustrates what the average score would have been for each examiner pair

Examiner Number	Good Candidate Overall Score			Average Candidate Overall Score			Weak Candidate Overall Score		
	Alone	Paired (avg)	Paired (agreed)	Alone	Paired (avg)	Paired (agreed)	Alone	Paired (avg)	Paired (agreed)
1	64	64	64	44	41	48	34	27	26
3	74	78	78	50	49	46	36	31	24
5	64	64	64	**38**	41	48	20	27	26
6	64	79	82	44	51	56	24	18	18
7	68	69	64	42	49	52	34	37	34
9	80	85	88	44	46	48	28	29	28
10	80	83	80	**34**	**34**	44	28	31	30
11	82	78	78	48	49	46	26	31	24
12	70	69	64	56	49	52	40	37	34
14	94	79	82	58	51	56	12	18	18
16	90	85	88	48	46	48	30	29	28
17	86	83	80	50	42	44	34	31	30
**Candidate** **Mean**	76.33 (10.54)	76.33 (8.19)	76 (9.87)	46.33 (6.86)	45 (6.41)	49 (4.33)	28.83 (7.69)	28.83 (6.27)	34 (5.46)
**Range**	30	21	24	24	17	12	28	19	16

*Avg* Average

**Fig. 1:**
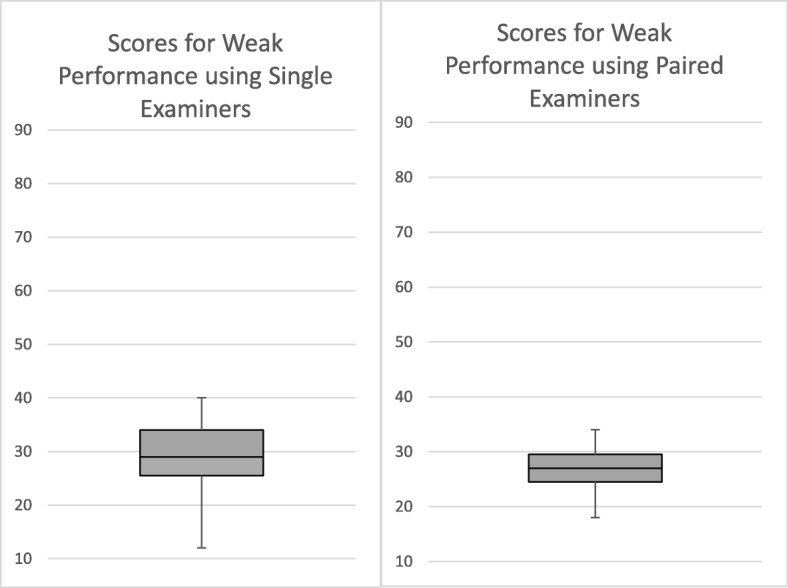
Box and Whisker Plots showing the Variability of Overall Scores for the Weak Performance using Single and Paired Examiners

**Fig. 2:**
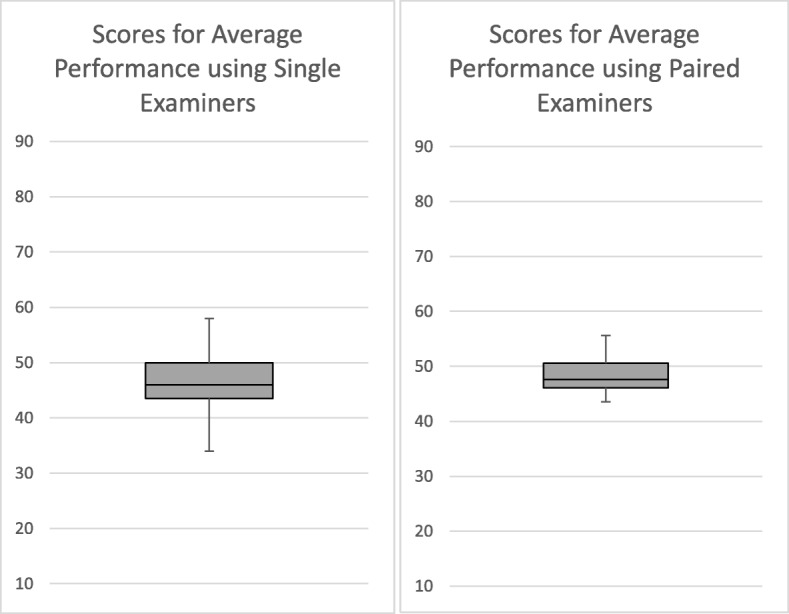
Box and Whisker Plots showing the Variability of Overall Scores for the Average Performance using Single and Paired Examiners

**Fig. 3:**
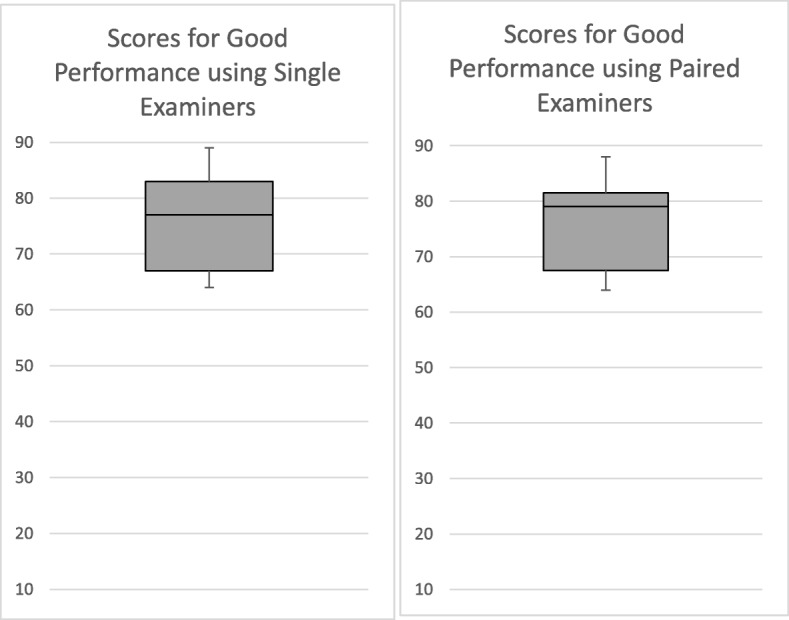
Box and Whisker Plots showing the Variability of Overall Scores for the Good Performance using Single and Paired Examiners

Table 1 also shows the overall scores awarded by examiners when in pairs and combining it with Figs. 1, 2 and 3 we can see that the ranges and standard deviations reveal that the variability between scores given by examiner pairs is, as might be expected, less than that in the assessment using 12 individual examiners.

Generalisability analysis allows for more in-depth analysis of the variance of our assessments, identifying the relative contribution of each of the components (or facets) of that assessment – the examiners (observations, O), the scenarios (S) and their interactions (SO). In the assessment using individual examiners, 87.1% of variance was found to be due to examiners while 12.9% was due to the interaction between the examiner and the scenario (Table 2).

**Table 2:** Analysis of Variance of the main facets of the assessment using 12 single examiners using EDU G Negative Variance was set to zero

Source	Components				
	df	MS	Random	%	SE
**O**	11	0.13392	0.04254	87.1	0.01752
**S**	2	0.00630	0.00000	0.0	0.00040
**OS**	22	0.00630	0.00630	12.9	0.00182
**Total**	35				100

*df* degrees of freedom*, MS* mean square*, SE* standard error, *O* Observations, *S* Scenarios, *SO* interaction of scenario and observation

### Reliability

We utilized both G-theory analysis and Classical Test Theory (CTT) analysis taking into consideration that many Schools of Medicine still refer to Cronbach’s Alpha as the measure for ‘reliability’. Using Classical Test Theory Cronbach’s alpha and intra-class correlation coefficients were calculated for the assessment using 12 single examiners and the second assessment using 6 examiner pairs. The reliability statistics for the two assessments were in fact comparable (Table 3).

**Table 3:** Reliability Statistics for the Assessments using both Single and Paired examiners

	Cronbach’s Alpha	Intraclass Correlation Co-efficient							
		Intraclass Correlation		95% Confidence Interval		F Test with True Value 0			
				Lower Bound	Upper Bound	Value	df1	df2	Sig
**Single Examiners**	0.99	Single Measures	0.887	.648	.997	98.97	2	22	.000
		Average Measures	0.990	.957	1.00	98.97	2	22	.000
**Paired Examiners**	0.983	Single Measures	0.925	.700	.998	60.533	2	10	.000
		Average Measures	0.987	.933	1.00	60.533	2	10	.000

*df* degrees of freedom

### Impact of pairing up on candidates’ score/outcome

We compared candidates scores when they were examined by 12 individual examiners with their scores when they were examined by 6 examiner pairs (see Table 1). The ‘good’ performance was awarded an honour by all 12 individual examiners and all 6 examiner pairs. Similarly, the weak performance was failed by all examiners – single and in pairs. However, when examined by individual examiners, the average performance was awarded 4 passes, 6 borderline results (between 40 and 49%) and failed by 2 examiners. When assessed by examiner pairs the average performance was not failed on any occasion but received 4 borderline marks and 2 passes. Wilcoxon signed rank test showed a statistically significant difference between mean scores for the average student (*p* = 0.0430).

### How each examiners’ marks changed when they were paired up

The marks given by each examiner when they examined singly were compared with the agreed mark given by the same examiner to each candidate when examining *in a pair*. The amount of change in each examiner’s overall mark for the three candidates was calculated. Table 4 shows the change in examiners marks and the direction of that change (a minus sign indicated their mark reduced when they paired up). The amount of change (regardless of whether positive or negative) for each examiner was calculated, representing the total amount of change in marks per examiner (bottom row Table 4).

**Table 4:** Changes in examiners’ marks when they moved from examining alone to examining in a pair

Examiners	Pair A		Pair B		Pair C		Pair D		Pair E		Pair F	
	1	5	3	11	7	12	6	14	9	16	10	17
Good	0.0	0.0	4	−4	−4	−6	18	−12	8	−2	0	−6
Average	4.0	10	−4	−2	10	−4	12	−2	4	0	10	−6
Weak	−8.0	6.0	−12	−2	0	−6	−6	6	0	−2	2	−4
**Total change**	12	16	20	8	14	16	36	20	12	4	12	16

There was a statistically significant negative correlation (− 0.808) between extroversion and change in examiners score - the higher an examiners’ score for extroversion the lower the degree of change in his or her score when paired up with a co-examiner (*p* = 0.001) (see Table 5).

**Table 5:** Relationship between the amount of change in examiners scores and personality. Only ‘Extroversion’ contributed significantly to the variation in marks per examiner with this personality score

	Spearman’s Correlation co-efficient rho	*P* value
**Neuroticism**	0.352	0.262
**Extroversion**	−0.808	0.001
**Openness to Experience**	−0.185	0.565
**Agreeableness**	−0.501	0.097
**Conscientiousness**	−0.451	0.141

## Discussion

This study showed acceptable and comparable reliability statistics for the assessment using both single and paired examiners. Using paired examiners there was less variability in candidate scores, which reflects that the process of reaching a consensus involves compromise and the impact of a ‘Hawk’ or ‘Dove’ is attenuated by a less stringent / more stringent examiner partner. The average performance was passed by all examiner pairs but failed by 2 examiners when marking individually. In high-stakes examinations this variability may have significant consequences on a candidate’s progression. Based on these observations we recommend the use of examiner pairs in high-stakes clinical assessments, such as final medical examinations, where judgements are made by 2 examiners and a final mark is reached by consensus.

Our results confirmed the findings of previous studies that in personality testing, doctors tend to score low for neuroticism and high for extroversion [23, 36]. They also suggest that a highly extrovert examiner is less likely to change their initial judgement when in discussion with his or her partner examiner; this could increase examiner variability and reduce the reliability of an assessment. This is perhaps not surprising as extroverts are described as assertive and talkative, two characteristics which would certainly enable an examiner to “stand their ground” as it were. Previous associations found between examiner stringency and extroversion (negative association) and neuroticism (positive association) were not repeated in this study [23]. As such definitive conclusions on possible association between personality factors and stringency cannot be drawn at this time.

The assessment team in a medical school will have data on the degree of stringency of existing examiners from previous candidate scorings awarded by them; the behaviour of new examiners, however, is largely unknown. Personality factor profiling of new examiners may assist in pairing them with existing examiners; this could, for example, avoid the risk of an extremely stringent examiner, who is also very extrovert, in negatively impacting the results of candidates marked by this examiner. Such a (new) examiner could, for example, be paired with a senior experienced examiner who is neither a ‘Hawk’ or a ‘Dove’. We recommend further studies to test for associations between personality factors and examiner stringency, and personality and size of change between an examiner’s independent score and the agreed score of an examiner pair.

### Limitations

Recruitment of participants proved difficult and so our sample was small and therefore statistical analysis might be compromised. There was a small number of female participants. It could be argued that there was a learning or testing effect in the set-up of our mock examination whereby the examiners assessed the same performances twice. Ideally, we would have used a larger number of video recordings to avoid compromising the internal validity of this study in this way however, increasing the length of the process would have made recruitment even more difficult.

Some investigators raised concerns about the recording of participants’ discussions giving rise to “the Hawthorne effect” where the awareness of being observed impacts on research participants’ behaviour [37] however, a review of the literature found very little empirical support for this effect in medical education [38].

## Conclusions

Our study shows that the practice of using paired examiners in clinical assessments has its merits. While using paired examiners may place greater demands on resources, in the case of high-stakes assessments and an increasingly litigious society, scores awarded by examiner pairs through discussion and consensus, are more resistant to variability, and may therefore be more easily defended in the case of appeals. Further studies on personality factors and examiner behaviour are needed to test associations and, depending on findings, whether personality testing may play a role in reducing examiner variability, and therefore improving the reliability of clinical examinations.

## Data Availability

The datasets used and/or analysed during the current study are available from the corresponding author on reasonable request.

## Abbreviations

CTT: Classical Test Theory
MRCP: Membership of the Royal College of Physicians
NEO-FFI: Neuroticism – Extroversion-Openness to experience Five Factor Index
OMIS: OSCE Management Information System Software (by Qpercom Ltd)
OSCE: Objective Structured Clinical Examinations
PACE: Practical Assessment of Clinical Examination Skills
SEM: Standard Error of Measurement
